# Supplementary material for: Immunomodulatory Effects of Stereotactic Body Radiotherapy and Vaccination with Heat-Killed Mycobacterium Obuense (IMM-101) in Patients with Locally Advanced Pancreatic Cancer
Source: Cancers (Basel). 2022 Oct 27;14(21):5299. doi: 10.3390/cancers14215299 (PMC9656492; doi:10.3390/cancers14215299)
Supplement: Supplementary file 1 [file cancers-14-05299-s001.zip › Supplementary figures.pdf]

Supplementary Figures

Supplementary Figure S1

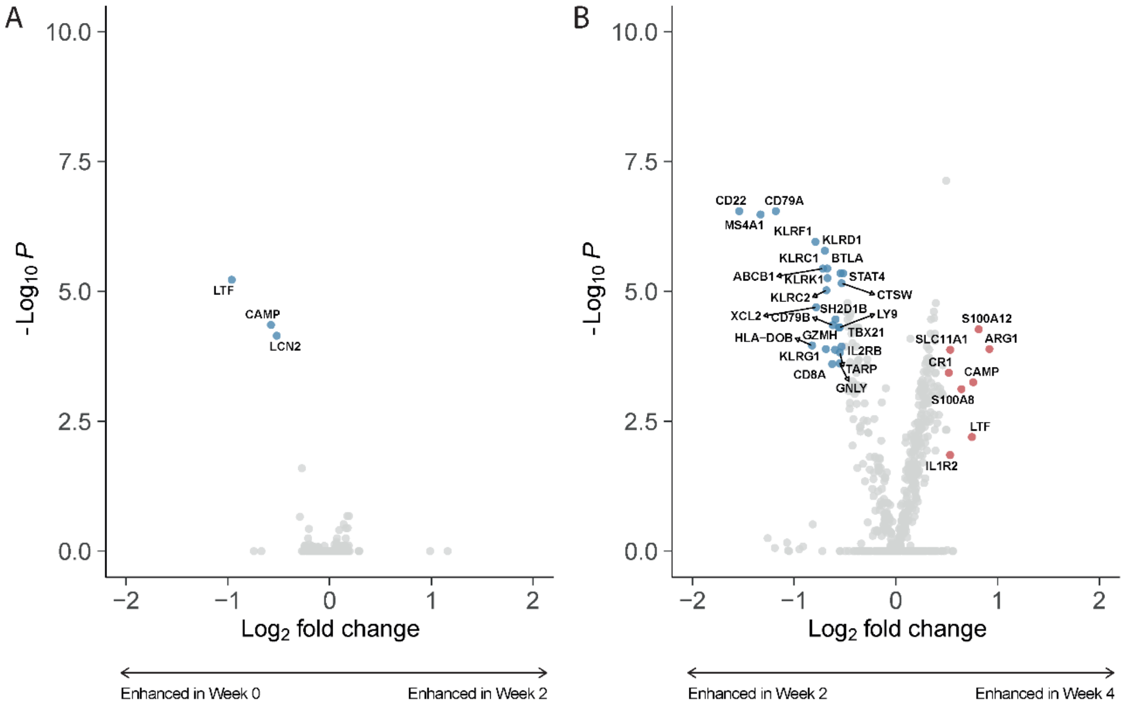

Supplementary Figure S1 One vaccination of IMM-101 has limited effect on immune-related gene expression. (A) Volcano plot demonstrating genes upregulated in week 2 compared to baseline (week 0). Highlighted genes have a log2fold change < 0.5 and p-value < 0.5. (B) Volcano plot demonstrating genes upregulated in week 4 compared to week 2. Highlighted genes have a log2fold change < or > 0.5 and p-value < 0.5.

Supplementary Figure S2

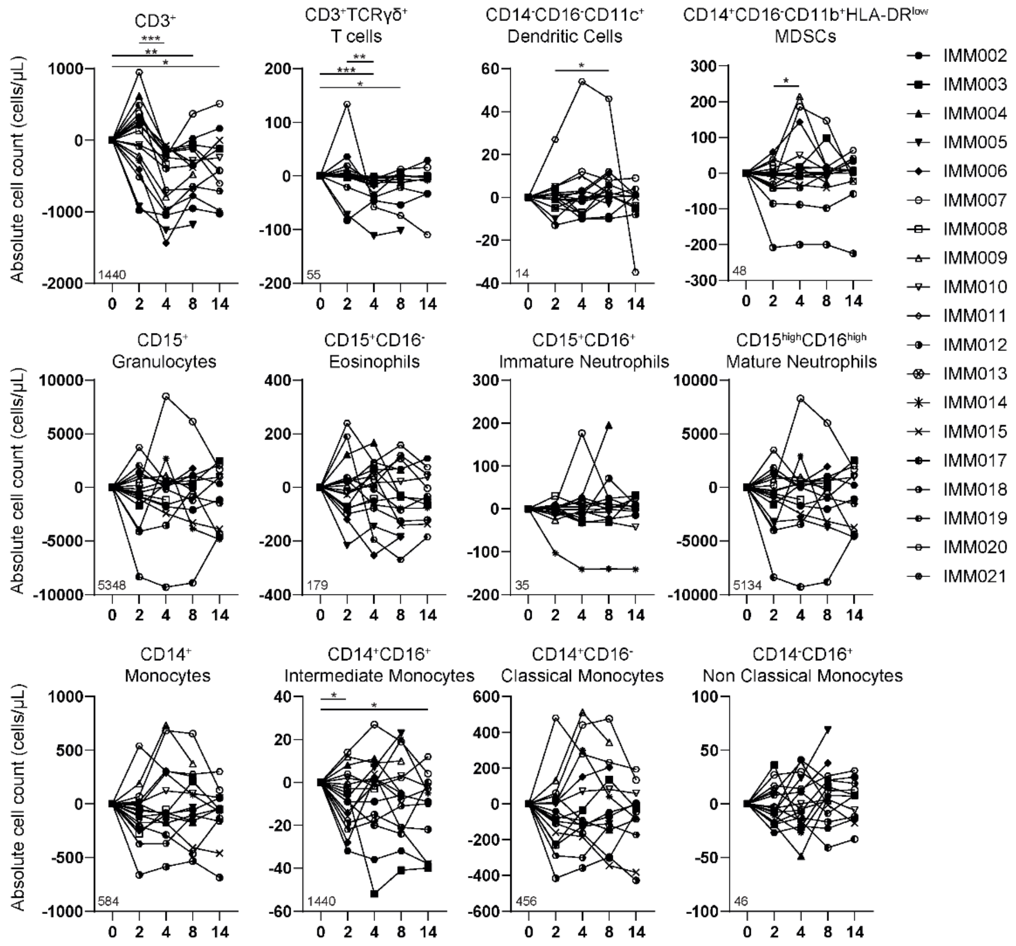

Supplementary Figure S2 SBRT/IMM-101 induced transient lymphodepletion. Number of CD3+, CD3+TCRγδ+, CD14−CD16−CD11c+, CD14+CD16−CD11b+HLA-DR^{low}, CD15+, CD15+CD16−, CD15+CD16+, CD15^{high}CD16^{high}, CD14+, CD14+CD16+, CD14+CD16− and CD14−CD16+ peripheral blood lymphocytes per µL blood. N = 19. Data were normalized for baseline (week 0) and paired per patient. Percentage in the bottom left corner is the average frequency at baseline. Significance was determined using the paired Wilcoxon signed-rank test. *p < 0.05, **p < 0.01, ***p < 0.0001.

Supplementary Figure S3

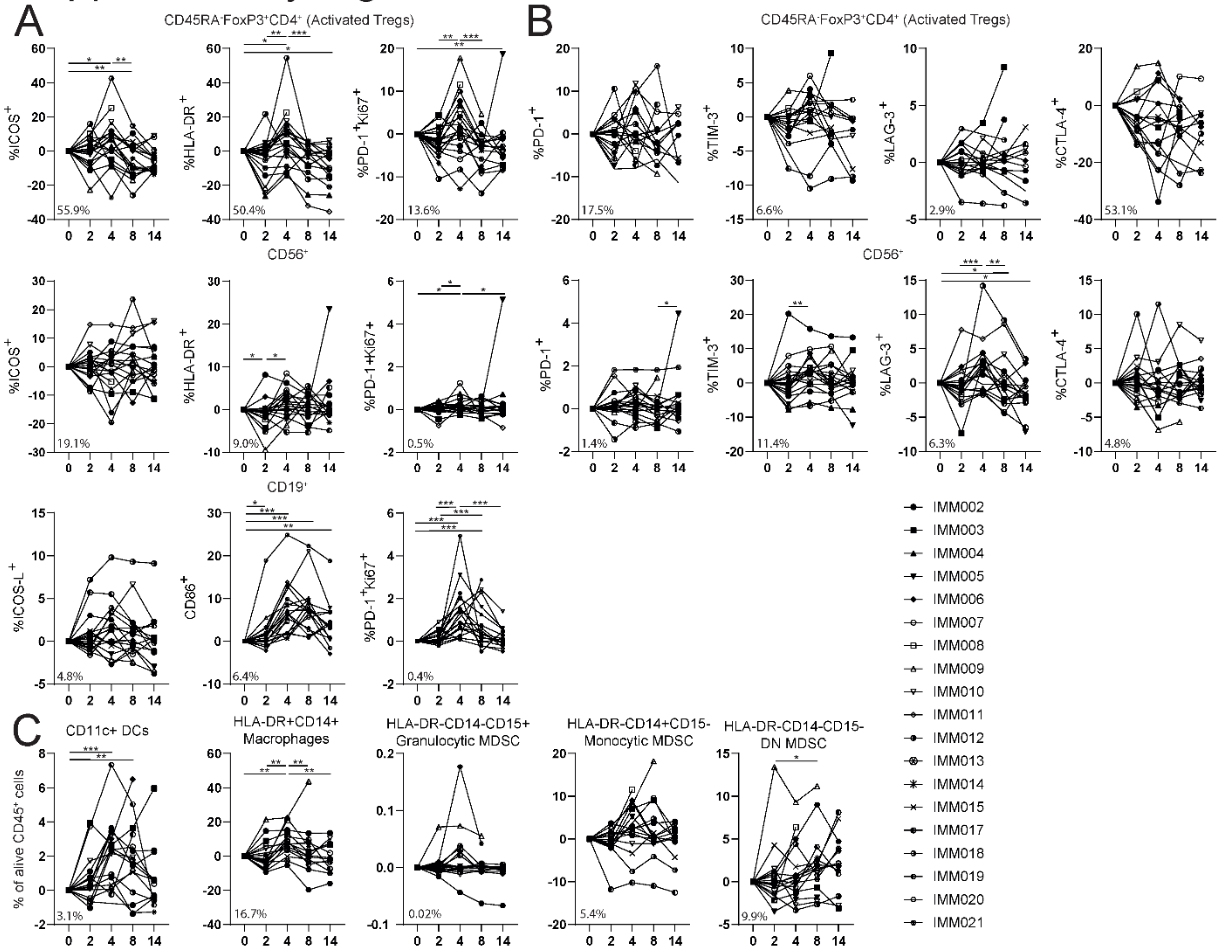

Supplementary Figure S3 SBRT/IMM-101 induced T-cell activation. (A) Percentage of ICOS+, HLA-DR+, PD-1+/Ki67+ subsets of CD45RA−FOXP3+CD4+ Tregs, CD56+ and CD19+ cells. (B) Percentage of PD-1+, TIM-3+, LAG-3, CTLA-4+ subsets of CD45RA−FOXP3+CD4+ Tregs and CD56+ cells. (C) Percentage of CD11+ dendritic cells, HLA-DR+CD14+ macrophages, HLA-DR−CD14−CD15+ granulocytic myeloid derived suppressor cells, HLA-DR−CD14+CD15− monocytic myeloid derived suppressor cells and HLA-DR−CD14−CD15− double negative myeloid derived suppressor cells compared to the total percentage of alive CD45+ cells. N =19. Data were normalized for baseline (week 0) and paired per patient. Percentage in the bottom left corner is the average frequency at baseline. Significance was determined using the paired Wilcoxon signed-rank test. *p < 0.05, **p < 0.01, ***p < 0.0001.
