# Supplementary material for: Immunomodulatory Effects of Stereotactic Body Radiotherapy and Vaccination with Heat-Killed Mycobacterium Obuense (IMM-101) in Patients with Locally Advanced Pancreatic Cancer
Source: Cancers (Basel). 2022 Oct 27;14(21):5299. doi: 10.3390/cancers14215299 (PMC9656492; doi:10.3390/cancers14215299)
Supplement: Supplementary file 1 [file cancers-14-05299-s001.zip › Supplementary tables.pdf]

Supplementary Table S1 All inclusion and exclusion criteria

**Inclusion criteria**	**Exclusion criteria**
Histologically confirmed pancreatic cancer, as indicated by a definite cytology report	Prior radiotherapy, chemotherapy other than FOLFIRINOX or pancreatic resection
Tumor considered locally advanced after diagnostic work-up including CT-imaging, using the DPCG criteria for locally advanced disease and diagnostic laparoscopy *	Second primary malignancy except in situ carcinoma of the cervix, adequately treated non-melanoma skin cancer, or other malignancy treated at least 5 years previously to diagnosis of pancreatic cancer and without evidence of recurrence
Age > 18 years and < 75 years	Current or previous treatment with immunotherapeutic drugs
European Cooperative Oncology Group (ECOG) performance status of 0 or 1	Previous allergic reaction to any mycobacterial product
American Society of Anesthesiologists (ASA) classification 1 or 2	Prolonged systemic corticosteroid or immunosuppressant medication use (i.e. >2 weeks)
No evidence of metastatic disease	Lymph node metastases from primary tumor outside the field of radiation
Largest tumor size < 7 cm x 7 cm x 7 cm<br>No direct tumor involvement of the stomach, colon or small bowel	Pregnancy, breast feeding<br>Serious concomitant systemic disorders that would compromise the safety of the patient or his/her ability to complete the study, at the discretion of the investigator
Normal renal function (Creatinine ≤ 30 ml/min)	Known history of Human Immunodeficiency Virus (HIV) (HIV-1/2 antibodies)
Normal liver tests (bilirubin < 1.5 times normal**; Alanine transaminase or aspartate transaminase < 5 times normal)	An active autoimmune disease that has required systemic treatment in past 2 years (i.e. with use of disease modifying agents, corticosteroids or immunosuppressive drugs). Replacement therapy (e.g., thyroxine, insulin, or physiologic corticosteroid replacement therapy for adrenal or pituitary insufficiency, etc.) is not considered a form of systemic treatment
Normal bone marrow function (White blood cell count > 3.0 x 10e9/L, platelet count > 100 x 10e9/L and hemoglobin > 5.6 mmol/l)	Diagnosis of immunodeficiency or receiving systemic steroid therapy or any other form of immunosuppressive therapy within 7 days prior to the planned first dose of the study. The use of physiologic doses of corticosteroids may be approved after consultation with the Sponsor
Ability to wear an Actiwatch device on non-dominant arm	Known active Hepatitis B (e.g., HBsAg reactive) or Hepatitis C (e.g., HCV RNA [qualitative] is detected)
Effective contraceptive methods	Live virus vaccine within 30 days of planned start of trial treatment

Written informed consent	Use of herbal remedies, including traditional Chinese herbal products (e.g., mistletoe)

** If patients are referred from an external hospital after completion of FOLFIRINOX chemotherapy, no diagnostic laparoscopy has to be performed. ** If bilirubin is higher than 35 umol/L, the placement of a metal biliary stent is mandatory.*
